# Advances in treatment and diagnosis of vision impairment

**DOI:** 10.1016/j.ebiom.2019.10.028

**Published:** 2019-10-30

On Sept 4, 2019, the National Health Service in England and Wales announced that voretigene neparvovec, a gene therapy for inherited vision loss, would become available for patients. Voretigene neparvovec is an adeno-associated viral vector-based gene therapy, delivered through a single retinal injection. It is designed for the treatment of patients with inherited retinal dystrophies caused by biallelic *RPE65* mutation. Affected individuals suffer progressive vision loss that can ultimately lead to total blindness. The treatment was first approved by the US Food and Drug Administration in late 2017, following completion of several clinical trials that showed functional improvement in vision. In a phase 3 trial, published by Russell and colleagues in *The Lancet* in 2017, 65% of participants who received the intervention showed maximum improvement in multi-luminance mobility testing at 1-year follow up (NCT#00999609).

This news follows an announcement of the first approved clinical trial of CRISPR-based medicine. AGN-151587 is designed to correct mutation in *CEP290,* a gene mutated in an inherited form of blindness named Leber congenital amaurosis 10. Developed as a collaboration between a pharmaceutical company (Allergan) and a genome editing company (Editas Medicine) in the USA, AGN-151587 will be evaluated for safety and efficacy in a phase 1/2 trial. It will be tested in multiple doses, but participants will each receive a single dosage as a subretinal injection following vitrectomy. The planned trial has already begun to enrol patients at the Massachusetts Eye and Ear Infirmary (Boston, MA, USA; NCT#03872479). The results will no doubt be of great interest, not just to vision researchers but also to the wider scientific community.

Therapies such as these are promising, but unfortunately do not offer a solution for all types of vision problems. These two treatments, for example, are designed to correct defects in specific genes. Vision loss, however, can arise for various reasons, including congenital disorders (eg, retinitis pigmentosa), age-related processes (eg, macular degeneration), or as a result of other conditions (eg, diabetic retinopathy). Additionally, for some treatments—such as voretigene neparvovec—there is an inherent requirement for the patient to still have sufficient retinal cells remaining. Stem cell therapies could circumvent these issues and offer new hope for patients even in later stages of vision loss. In July, 2019, a woman in Japan was the first person to receive a corneal transplant made from induced pluripotent stem cells. The intervention has reportedly restored clear vision to the woman. The work, led by Kohji Nishida from Osaka University (Osaka, Japan), is the first in a series of four operations that will be done. Although the initial news report is encouraging, further follow-up will reveal whether there are any adverse events and assess whether the vision improvement is maintained in the long term.

These therapies represent the culmination of many years of research to develop novel treatment strategies. For some forms of vision impairment, however, no innovative therapy is required. A simple corrective measure, such as glasses (for myopia) or ocular surgery (for cataracts), is already available. According to a report released this year by WHO, an estimated 285 million people globally have vision impairment. Of these, 80% are considered to have an avoidable blindness—42% need glasses and 33% require treatment for cataracts. The WHO report highlights inadequate access to health-care diagnostics and treatment as a major barrier in eliminating avoidable blindness. Here, novel digital health solutions could help overcome these barriers by supporting diagnosis and referral.

In a trial published in July, 2018, in *The Lancet Global Health*, Andrew Bastawrous and colleagues from the London School of Hygiene & Tropical Medicine (London, UK) assessed the utility of a mobile phone-based screening system called Peek Vision in schools in Trans Nzoia, Kenya. Centred around a smart-phone app (Peek Acuity), the system aims to identify and refer those who need examination by an optometrist. Peek Acuity involves a modified Snellen vision test and can be done quickly by a teacher in a classroom setting. In the trial, the guardians of the children requiring a referral were given an information card and received a follow-up reminder via short message service (SMS). The authors found that 54% of children who required a referral for vision impairment attended versus 22% of children who were assessed by conventional screening. Bastawrous’ team is now developing Peek Retina, a portable ophthalmoscope that attaches to a smartphone camera. It can be used to image a dilated retina, enabling screening of the optic nerve for pathologies such as glaucoma or diabetic retinopathy.

Analysis of any retinal scan, however, requires specialists. In an article published this year in the *British Journal of Ophthalmology*, the International Council of Ophthalmology reports a mean of just 3·7 ophthalmologists per million people in low-income countries (*vs* a mean 76·2 ophthalmologists per million people in high-income countries). The growing burden of an ageing population in middle-income and high-income countries is also expected to place a strain on medical resources. Machine-learning approaches have increasingly been used to automate analysis of 2D images, but interpretation of clinically heterogenous images—such as the 3D optical coherence tomography (OCT) scans usually obtained when assessing a patient's eye—represent an additional challenge.

In a study published in September, 2018, in *Nature Medicine*, a deep-learning framework developed by experts from Moorfields Eye Hospital (London, UK) and Google's DeepMind (London, UK) was described. The analysis framework is based on two neural networks. Firstly, a segmentation network analyses the OCT scan to highlight pathological features present, which is then interpreted by a classification network that outputs a diagnosis and referral recommendation. A referral assessment (urgent, semi-urgent, routine, or observation) based on the most urgent diagnosis identified on the scan is provided with a confidence prediction. The system is reportedly able to provide recommendations for more than 50 pathologies commonly affecting the eye, with performance matching or exceeding clinical experts.

Integration of such approaches into routine clinical care could facilitate timely screening and early enough intervention for patients, meaning that some could avoid reaching late-stage vision loss altogether. *EBioMedicine* seeks to be a platform for publication of the full breadth of scientific work that underpins such advances ultimately reaching the clinic and offering hope to those affected by vision loss. Related articles already published by *EBioMedicine* are available to read Online in our Sensory Systems Research collection.

*EBioMedicine*

